# Lost in the Woods: A Case Report of a Radiolucent Wooden Foreign Body in a Child After Negative CT, Detected on Repeat Ultrasound

**DOI:** 10.7759/cureus.112961

**Published:** 2026-07-19

**Authors:** Stephanie L Eswine, Todd P Chassee, Chris A Benner, Jeffrey S Jones

**Affiliations:** 1 Emergency Medicine, Corewell Health - Michigan State University Emergency Medicine Residency Program, Grand Rapids, USA; 2 Emergency Department, Michigan State University College of Human Medicine, Grand Rapids, USA; 3 Pediatric Emergency Medicine, Helen DeVos Children's Hospital, Grand Rapids, USA

**Keywords:** case report, emergency medicine, foreign body, pediatrics, point‑of‑care ultrasound, ultrasound, wooden

## Abstract

Retained soft-tissue foreign bodies are a common but often missed complication of traumatic wounds, particularly when composed of radiolucent materials such as wood, which can evade detection on standard imaging. Although radiography and computed tomography (CT) are highly sensitive for radiopaque objects, they may fail to identify organic foreign bodies. This makes timely diagnosis challenging and underscores the importance of ultrasound when the mechanism suggests wood. A previously healthy five-year-old boy presented to a community emergency department after sustaining a puncture wound to the right thigh from a tree branch, with localized tenderness but no obvious foreign body on examination. Plain radiographs of the femur and pelvis and a CT of the pelvis demonstrated subcutaneous air but no retained foreign body; the wound was explored and irrigated, and the patient was discharged on oral antibiotics. He then presented to a children’s hospital with worsening pain, erythema, and purulent drainage. Initial ultrasound showed a small, heterogeneous subcutaneous collection concerning for an abscess, and he improved transiently with intravenous, then oral, clindamycin. Within 24 hours, he returned with recurrent pain, and repeat ultrasound revealed a linear echogenic structure within the deep subcutaneous tissue consistent with a wooden foreign body, associated with an adjacent abscess. Operative exploration led to the removal of a 6-cm piece of tree branch from the thigh, with subsequent uncomplicated recovery. This case illustrates that CT may not exclude a retained wooden foreign body when the mechanism of injury strongly suggests organic material. In patients with persistent pain, swelling, drainage, or recurrent symptoms, repeat targeted ultrasound should be considered even after initially negative imaging.

## Introduction

Retained foreign bodies are a well-recognized complication of soft tissue injuries and can lead to infection, delayed healing, chronic pain, and repeated healthcare encounters if not promptly identified and removed [1]. In children, puncture wounds sustained during play and outdoor activities are common, and organic materials such as wood and plant thorns are frequent culprits. Because wood is typically radiolucent, however, it may be difficult to detect on standard imaging, and both plain radiographs and computed tomography (CT) can fail to reveal wooden fragments, particularly in the acute setting when attenuation may resemble air or soft tissue [2].

Ultrasound has emerged as an important modality for detecting radiolucent foreign bodies in soft tissue, especially wooden objects [2]. When the appropriate technique is used, ultrasound can detect soft tissue foreign bodies, including wood, with sensitivities and specificities commonly reported in the range of roughly 70-90% and 90-97%, respectively, substantially outperforming CT for radiolucent materials [3,4]. Wood splinters often appear as linear echogenic structures with posterior acoustic shadowing and a surrounding hypoechoic halo corresponding to edema or abscess [3]. Bedside ultrasound allows dynamic, focused assessment directly over the area of maximal tenderness without radiation exposure, making it particularly advantageous in pediatric patients [4-6]. More broadly, point-of-care ultrasound has become an important emergency department tool because it can provide rapid bedside diagnostic information and support time-sensitive clinical decisions across different ED scenarios [7]. Despite these strengths, ultrasound may be delayed or not repeated when negative radiography or CT provides false reassurance. This is clinically important because the sonographic appearance of an organic foreign body may become more conspicuous as edema, abscess, or inflammatory change develops.

This case is notable in that both CT and the initial ultrasound were nondiagnostic for a sizable wooden foreign body, whereas repeat ultrasound became diagnostic only after clinical progression and evolution of the local inflammatory response. This sequence underscores the broader value of bedside ultrasound in emergency department workflows, where mechanism‑guided, repeat sonography can provide rapid, focused information at the point of care when symptoms evolve after initially negative imaging.

We present the case of a five-year-old boy with a thigh puncture wound from a tree branch in whom radiography, CT, bedside ultrasound, and initial wound exploration failed to identify a retained wooden foreign body. He re-presented with worsening symptoms, and a repeat ultrasound ultimately detected a sizable, retained fragment that was removed surgically. This case highlights that, in pediatric puncture wounds involving wood, negative radiography, negative CT, and even an initially nondiagnostic ultrasound should not exclude a retained foreign body when clinical findings persist or progress. The case supports a mechanism-guided approach with early and repeat targeted ultrasound when organic material is suspected.

## Case presentation

A previously healthy five-year-old boy presented to a community emergency department after sustaining a puncture wound to the right thigh when he jumped from a tree and was impaled by a tree branch. On initial evaluation (day 0), vital signs were within normal limits (blood pressure 117/88 mmHg, heart rate 80 beats/minute, respiratory rate 28 breaths/minute, temperature 36.8 °C). Elevated blood pressure was attributed to pain and distress at the time of examination and was not felt to represent sustained hypertension. Examination showed a small puncture wound and abrasion of the right thigh with localized tenderness; no foreign body was palpable on bedside assessment. Distal pulses, capillary refill, and sensation were intact, and the limb was warm and well perfused. He was able to bear weight with mild discomfort, and hip and knee range of motion were preserved, limited by pain at the wound site. There were no clinical signs of joint effusion, compartment syndrome, or deep‑space infection, and the wound trajectory was estimated to be superficial within the subcutaneous tissues. 

**Figure 1:**
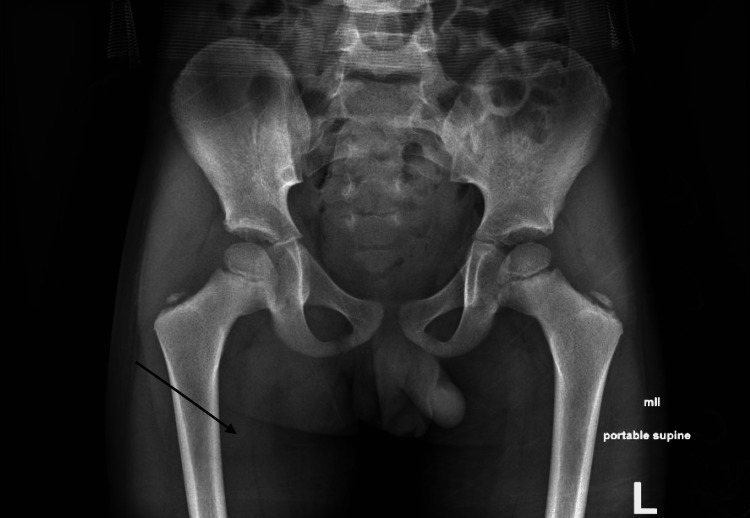
Plain radiograph of the pelvis Anteroposterior radiograph of the pelvis and right femur obtained at the initial emergency department visit without a visible foreign body or osseous injury. The arrow marks the site of the puncture wound.

Plain radiographs of the right femur and pelvis were obtained and did not reveal a foreign body or osseous injury (Figure 1). Because he remained significantly uncomfortable, computed tomography (CT) of the pelvis was performed, which demonstrated subcutaneous air in the right inguinal region without a definable foreign body. At this community emergency department, CT was readily available and commonly used to evaluate deep soft tissue injury, whereas point‑of‑care ultrasound for foreign body detection was not yet integrated into routine practice, so ultrasound was not obtained during the index visit. Magnetic resonance imaging (MRI) was not available. The wound was explored and probed at the bedside to a depth greater than 1 cm and irrigated with saline and chlorhexidine solution. He was discharged home on oral amoxicillin-clavulanate (e.g., 40-45 mg/kg/day of the amoxicillin component divided twice daily), chosen to provide broad coverage for contaminated soft tissue injury, including common skin flora and β‑lactamase-producing organisms after an outdoor puncture wound.

**Figure 2:**
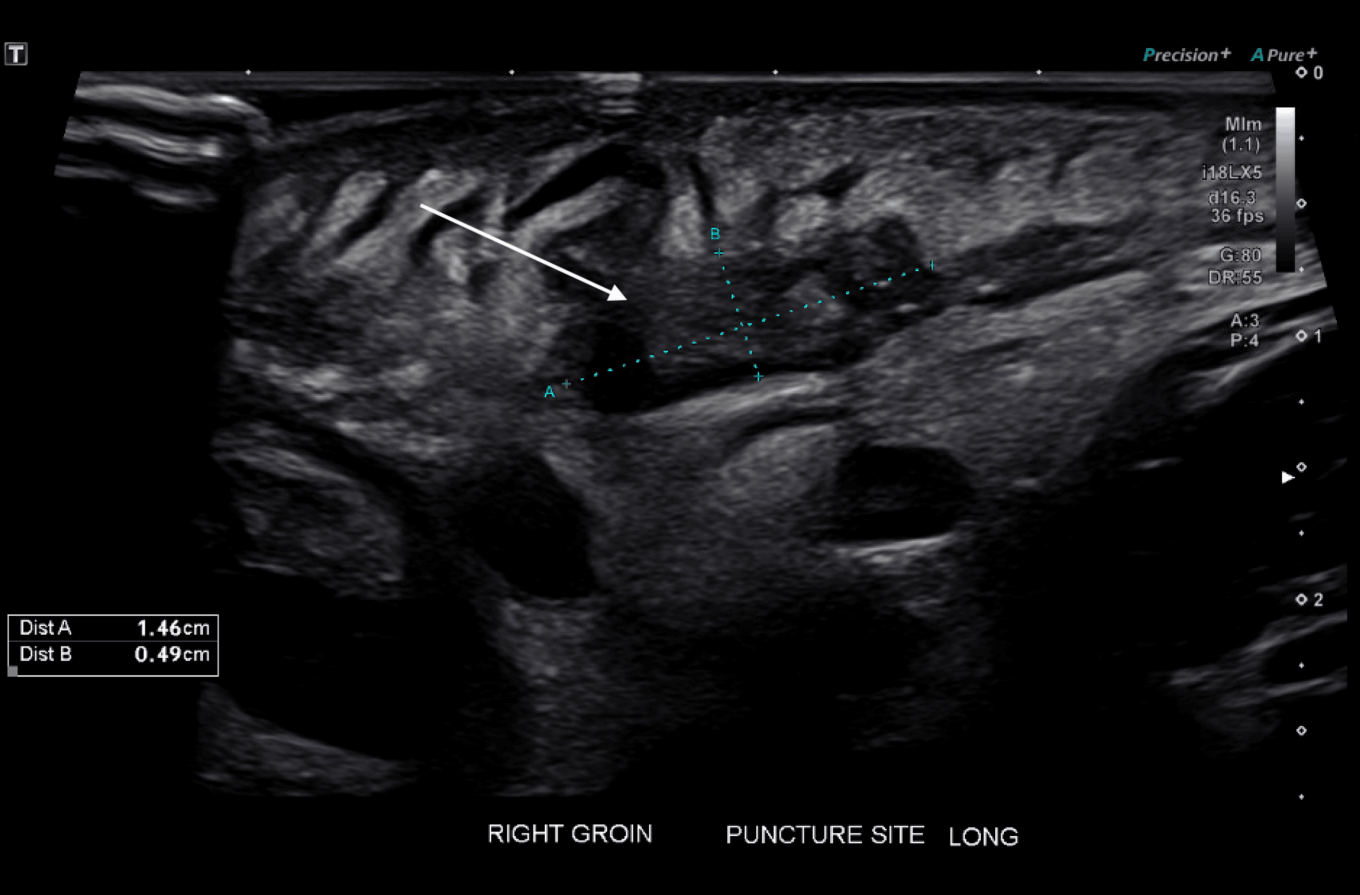
Longitudinal ultrasound of the right thigh (day 8) Linear heterogeneous lobular subcutaneous collection measuring 2.2 × 1.5 × 0.5 cm concerning for abscess (arrow), without a clearly visualized foreign body. The image was acquired using a high‑frequency linear transducer (e.g., 10-15 MHz) at a depth of approximately 3-4 cm with focal zones placed over the area of maximal tenderness.

Eight days later (day 8), he presented to a children’s emergency department with worsening pain and purulent drainage despite outpatient antibiotics. Examination demonstrated erythema and induration extending from the wound toward the right inguinal region, marked tenderness, and active purulent drainage. Laboratory evaluation showed a normal white blood cell count (8.2 ×10³/µL) without leukocytosis, a C‑reactive protein of 5 mg/L, and an erythrocyte sedimentation rate of 6 mm/hr, all within pediatric reference ranges. Ultrasound of the right thigh, performed by pediatric radiology using a high‑frequency linear transducer, demonstrated a heterogeneous lobular subcutaneous collection measuring 2.2 × 1.5 × 0.5 cm concerning for an abscess. No definite foreign body was visualized. The examination was targeted to the area of maximal tenderness and along the presumed wound tract, but pain, limited cooperation, and small pockets of overlying gas modestly restricted visualization of deeper structures (Figure 2). He received intravenous clindamycin (e.g., 10-13 mg/kg every 8 hours), selected to improve coverage for gram‑positive and anaerobic organisms in the setting of worsening cellulitis and an evolving abscess, and was admitted overnight for intravenous antibiotics, monitoring, and possible operative intervention. Once his examination improved, he was transitioned to oral clindamycin at standard pediatric dosing (approximately 10-13 mg/kg every 8 hours) to complete a seven‑day course of therapy for soft tissue infection. 

**Figure 3:**
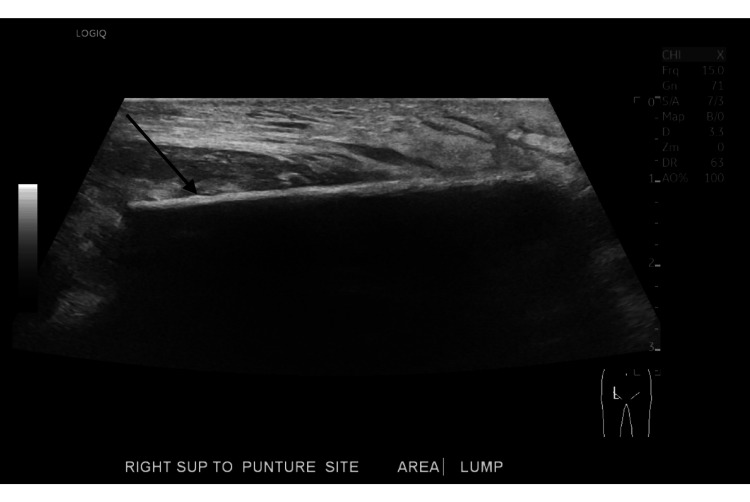
Ultrasound demonstrating retained wooden foreign body (day 10) Longitudinal ultrasound image of the right thigh obtained on the third presentation, showing a linear echogenic structure (arrow) within the deep subcutaneous tissues with posterior acoustic shadowing and an adjacent fluid collection consistent with abscess. The image was acquired using a high‑frequency linear transducer (e.g., 10-15 MHz) at a depth of approximately 3-4 cm.

On hospital day 1 (day 9), his examination appeared improved, and no fluctuance was appreciated, so he was discharged on oral clindamycin. He returned within 24 hours (day 10) with recurrent, worsening pain. Repeat ultrasound of the right thigh, again performed by pediatric radiology using a high‑frequency linear transducer and targeted to the wound tract and area of maximal tenderness, revealed a linear echogenic structure within the deep subcutaneous tissues measuring 4.9 × 0.9 cm, with an associated abscess measuring 4.5 × 0.5 × 1 cm. At this time, improved patient cooperation and a more mature fluid collection enhanced contrast, allowing clearer visualization of the retained wooden foreign body (Figure 3).

**Figure 4:**
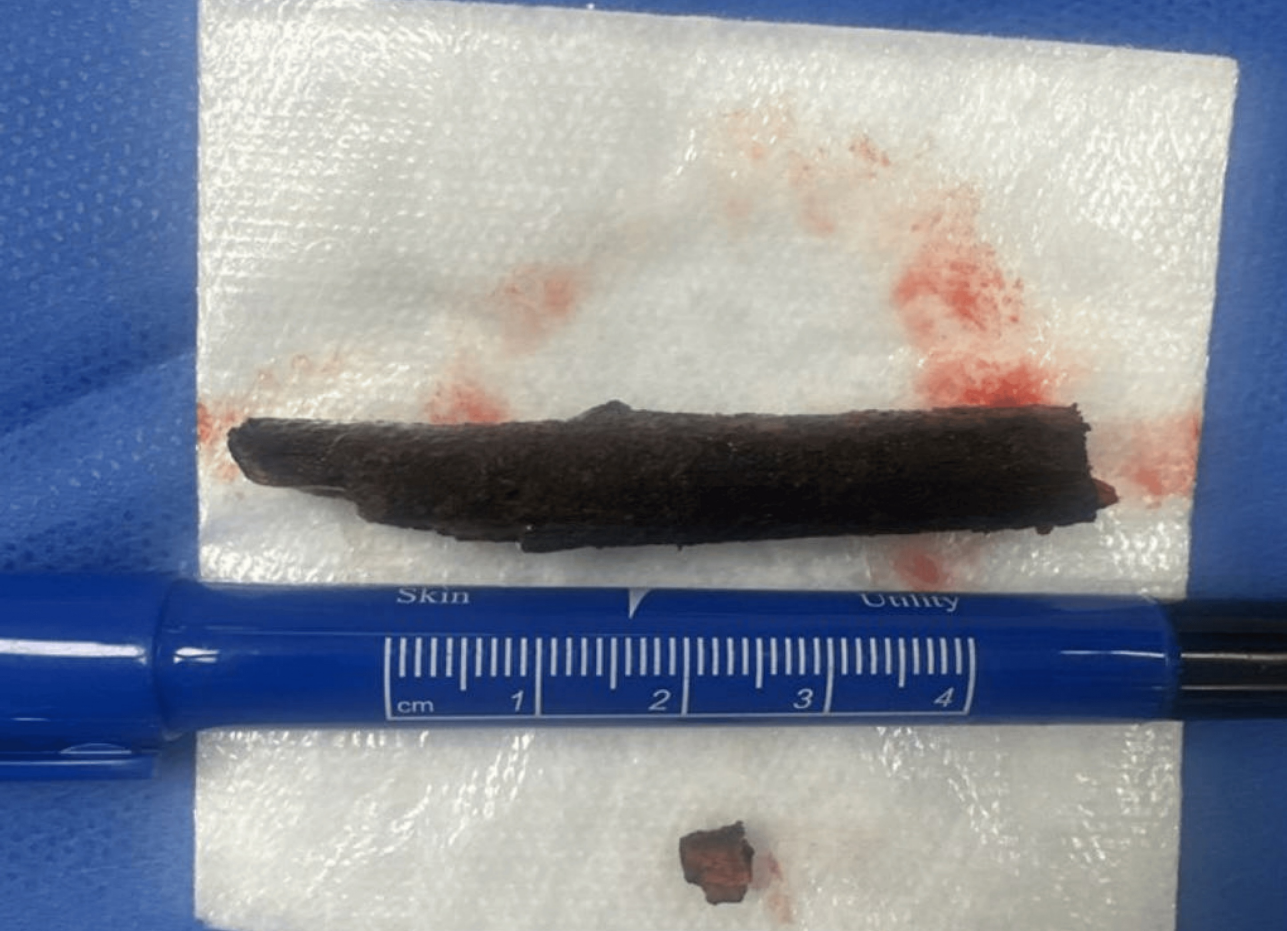
Removed wooden foreign body Intraoperative photograph of the extracted wooden foreign body, a tree branch fragment measuring approximately 6 cm in length and 1 cm in diameter, removed from the right thigh.

Given persistent pain and repeat ultrasound evidence of a linear echogenic foreign body with an associated abscess, pediatric general surgery performed operative wound exploration on day 10, six hours after the ED return visit. An incision was made over the area of maximal tenderness, and dissection through the subcutaneous tissues revealed an abscess cavity. Incision and drainage were performed, purulent fluid was expressed, and a wooden fragment measuring approximately 6 cm in length and 1 cm in diameter was removed in two pieces (Figure 4). The wound tract was probed and irrigated to exclude additional fragments, and no further foreign material was identified. Blood cultures showed no growth, and intraoperative wound cultures were not obtained.

Following removal of the retained wooden foreign body, he recovered without further reported complications. At the one-week follow‑up in the surgery clinic, he had no recurrent pain, drainage, erythema, or other reported complaints. The overall course is summarized in Table 1. 

**Table 1:** Timeline of clinical course

Time Point	Clinical Findings	Imaging/Interventions	Disposition
Day 0 (Initial presentation)	Puncture wound from a tree branch to the right thigh; local tenderness; small puncture wound with an overlying abrasion; 1-cm right inguinal lymph node	Plain radiographs of the femur and pelvis without foreign body or osseous injury; computed tomography of the pelvis with subcutaneous air in the right inguinal region without a definable foreign body.	Continued evaluation
Day 0 (Same visit)	Wound exploration and irrigation performed; probed to depth >1 cm	Bedside wound exploration and irrigation with saline and chlorhexidine	Continued evaluation
Day 0 (Discharge)	Discharged on oral amoxicillin-clavulanate	None	Home with antibiotics
Day 8 (Second presentation)	Worsening pain and purulent drainage despite antibiotics; erythema and induration extending toward the inguinal region	Ultrasound showing a 2.2 × 1.5 × 0.5 cm heterogeneous lobular subcutaneous collection concerning for abscess, without a clearly visualized foreign body.	Admitted for intravenous antibiotics and monitoring
Day 9 (First admission)	Examination improved; no fluctuance appreciated	Received intravenous clindamycin; evaluated by pediatric surgery	Observation overnight
Day 9 (Discharge)	Discharged on oral clindamycin	None	Home with oral antibiotics
Day 10 (Third presentation)	Worsening right thigh pain despite antibiotic treatment.	Repeat ultrasound: linear foreign body 4.9 × 0.9 cm with associated abscess 4.5 × 0.5 × 1 cm	Scheduled for surgery
Day 10 (Operative intervention)	Removal of 6-cm tree branch and associated abscess	Operative exploration by pediatric surgery; successful foreign body removal	Uncomplicated recovery

## Discussion

Retained foreign bodies are a well-recognized but frequently underdiagnosed complication of soft tissue injuries, particularly in the emergency setting [3]. In children, puncture wounds from wooden objects are common during outdoor play, yet wooden foreign bodies are radiolucent and often not apparent on standard radiographs. Failure to identify a retained fragment can lead to delayed diagnosis, recurrent symptoms, infection, and repeated healthcare encounters, as seen in this case.

Wooden foreign bodies present specific imaging challenges. Radiographs and computed tomography (CT) are highly sensitive for radiopaque materials such as metal and glass, but they are substantially less reliable for wood, which may attenuate similarly to soft tissue or gas depending on hydration and orientation [8]. CT has a specificity of approximately 93-98%, but sensitivity ranges roughly 63-79% and can drop much lower for soaked, tiny, or anatomically difficult wood [6,8,9]. Peterson et al. and others have shown that the CT appearance of wood is time‑dependent: freshly embedded, relatively dry wood can appear denser, whereas waterlogged or chronically desiccated wood may become indistinguishable from surrounding soft tissues or air [9]. When the mechanism of injury clearly involves wood, a negative CT should not be considered definitive. Instead, imaging must be interpreted within the broader clinical context, and persistent pain, swelling, or drainage should prompt reassessment for a retained organic foreign body.

Ultrasound is a key modality for detecting radiolucent foreign bodies, including wood. In cadaver feet scanned by musculoskeletal radiologists, overall sensitivity was 90.0% and specificity 96.7% for wooden fragments [6]. Point‑of‑care ultrasound (POCUS) by non-radiologists still detects wood reasonably often, but specificity is less consistent. Nurse practitioners had 95% sensitivity for wood after brief training, yet overall specificity for mixed foreign bodies was only 50% [10]. Across studies of soft tissue foreign bodies, POCUS appears to have pooled sensitivities and specificities of approximately 72% and 92%, respectively [4]. Accuracy varies most predictably with foreign body size and tissue depth, and ultrasound performs best for superficial foreign bodies at least ~2 mm in size, becoming less reliable for very small or deeper targets [6,9-11]. Jacobson et al. and others have described patients in whom wooden foreign bodies were missed on radiography or CT but subsequently identified by ultrasound, often appearing as linear hyperechoic structures with posterior acoustic shadowing and a surrounding hypoechoic halo corresponding to edema, inflammatory change, or abscess [6,9-11]. Because ultrasound allows focused bedside assessment over the area of maximal tenderness and avoids radiation exposure, it is particularly well suited for pediatric patients. In our case, radiography and CT were nondiagnostic for a sizable wooden fragment, whereas repeat ultrasound - performed after further evolution of the inflammatory response - revealed the characteristic linear echogenic foreign body with an associated abscess, adding a pediatric example that reinforces the value of mechanism‑guided, repeat ultrasound when initial sonography is nondiagnostic [4,11].

In our case, the CT at the initial community emergency department visit demonstrated subcutaneous air but no definable foreign body, and bedside wound exploration did not reveal a retained fragment. CT was selected after negative radiographs because it was readily available and commonly used locally to evaluate deep soft tissue injury, whereas point‑of‑care ultrasound for foreign body detection was not yet embedded in routine emergency practice. At the tertiary children’s hospital, initial ultrasound identified a small heterogeneous subcutaneous collection consistent with an early abscess but did not clearly demonstrate a foreign body. Repeat ultrasound on the patient’s return visit, after further evolution of the inflammatory response, revealed a classic linear echogenic structure compatible with a wooden fragment and an associated abscess, prompting operative exploration and definitive management. This sequence underscores that ultrasound is both an initial diagnostic tool and a valuable follow‑up modality, with its yield potentially increasing over time as edema and fluid collections develop.

On repeat ultrasound, the foreign body was measured at approximately 4.9 × 0.9 cm, whereas the surgically retrieved specimen measured about 6 × 1 cm. This discrepancy likely reflects underestimation by ultrasound, as acoustic shadowing, partial insonation, and irregular fragment orientation can obscure true margins and cause wooden foreign bodies to appear smaller than their actual dimensions [4,6,11]. Clinicians should interpret sonographic measurements as approximations and rely on operative exploration for definitive assessment of size and extent.

This clinical course highlights a diagnostic safety issue: clinicians may anchor on negative early imaging despite a mechanism that remains highly suspicious for a radiolucent organic foreign body. In pediatric puncture wounds involving wood, persistent pain, recurrent symptoms, purulent drainage, or evolving inflammatory findings should trigger repeat focused ultrasound or surgical reassessment rather than continued reliance on the initial negative CT or nondiagnostic ultrasound. From the initial presentation, the history of a puncture wound from a wooden object should have raised concern for a retained wooden fragment. Despite negative radiography, CT, and bedside exploration - and in the setting of limited MRI availability - the child's persistent pain and later development of erythema and purulent drainage were clear signs of ongoing pathology. When wood or other organic material is involved, clinicians should favor early ultrasound, maintain a low threshold to repeat ultrasound if symptoms persist, and avoid relying on CT alone to exclude a retained foreign body [4]. Bedside ultrasound, in particular, can provide rapid, focused reassessment in the emergency department, reducing dependence on delayed or less informative imaging and accelerating decisions about continued observation versus operative source control [9].

Several systems‑level lessons also emerge. Emergency clinicians should not assume that advanced imaging such as CT is definitive in all cases; understanding modality‑specific strengths and limitations is essential. Incorporating POCUS into routine workflows for soft tissue injuries allows clinicians to obtain immediate, targeted information at the bedside, supporting serial reassessment and dynamic decision‑making when symptoms evolve after initially negative imaging [10,11]. At the institutional level, training, protocols, and equipment availability can either facilitate or impede the use of bedside ultrasound for suspected radiolucent foreign bodies. Discharge instructions should explicitly advise patients and caregivers to return promptly if pain, swelling, drainage, or systemic symptoms worsen, and clinicians should document any ongoing concern for a possible retained foreign body in the discharge note, so that subsequent providers recognize the residual diagnostic uncertainty [11]. Clear documentation of diagnostic uncertainty, explicit return precautions, and planned reassessment are critical when a retained foreign body cannot be confidently excluded. Early involvement of surgical consultants may also be warranted when bedside exploration is limited by depth, patient discomfort, or anatomic location, particularly in children. In this case, collaboration between emergency physicians, radiology, and pediatric surgery, combined with repeat ultrasound guided by mechanism of injury and evolving symptoms, ultimately led to successful removal of a sizable wooden branch and an uncomplicated recovery.

This report describes a single pediatric case from two hospitals and cannot establish definitive comparative performance between CT and ultrasound for wooden foreign bodies. The imaging findings and diagnostic sequence reflect local practice patterns, operator experience, and resource availability at the time of care and may not generalize to all emergency settings or patient populations. Quantitative data on CT sensitivity/specificity and ultrasound performance are drawn from prior experimental and clinical studies; our case illustrates how these constraints can play out in practice but should be interpreted as hypothesis‑generating rather than confirmatory. Finally, some examination and laboratory details were reconstructed from documentation rather than prospectively standardized, which may limit the granularity of clinical descriptors and antibiotic decision‑making.

## Conclusions

This case demonstrates that negative plain radiography and computed tomography do not reliably exclude radiolucent wooden foreign bodies, particularly in pediatric puncture wounds involving organic material. Clinicians should let the mechanism of injury and the evolving clinical course guide imaging decisions, prioritizing early ultrasound when wood is suspected and repeating ultrasound if pain, swelling, or drainage persist despite initially negative studies. Recognizing that the yield of ultrasound may increase as inflammation and associated fluid collections evolve is especially important when the initial sonographic evaluation is nondiagnostic. Maintaining clinical suspicion, involving surgical consultants when bedside exploration is limited, and providing clear return precautions may help reduce diagnostic delay in similar cases, but these strategies cannot be assumed to definitively prevent complications or repeated emergency visits. Repeat ultrasound and continued vigilance for a retained foreign body should be viewed as practical measures that may improve timely diagnosis and outcomes in comparable presentations rather than as proven guarantees.
